# Supplementary material for: Are carbon emissions trading and green financial instruments synergistic? -Comprehensive quantitative research based on content analysis
Source: PLoS One. 2024 Mar 7;19(3):e0298601. doi: 10.1371/journal.pone.0298601 (PMC10919601; doi:10.1371/journal.pone.0298601)
Supplement: S1 Data — (ZIP) [file pone.0298601.s001.zip › Data/地级市Z_F工作报告.zip!/╕ú╜¿/╚¬╓▌╩╨/└┤╫╘╣1⁄2╓┌║┼í╛┬φ┐╦╩2╛▌═°í┐_2006.docx]

各位代表：

　　现在，我代表泉州市人民政府向大会作工作报告，请予审议，并请政协委员和其他列席人员提出意见。

　　一、过去六年政府工作回顾

　　本届政府的六年，跨越了“十五”五年和“十一五”开局之年。在市委领导下，我市以邓小平理论和“三个代表”重要思想为指导，深入贯彻党的十六大精神，树立和落实科学发展观，推进和谐社会建设，主动融入海峡西岸经济区建设大局，全面建设小康社会，努力克服发展中的各种困难，“十五”经济社会发展取得辉煌成就，“十一五”经济社会发展实现良好开局。

　　综合经济实力持续增强。六年来，我们致力突破经济运行中的基础性、方向性、关键性问题，经济增长质量和效益不断提高。全市生产总值从2000年的931.08亿元增加到2006年的1900亿元（预计，下同），继续保持全省首位，年均增长12.8％；财政总收入从2000年的57.52亿元增加到2006年的185亿元，年均增长21.5％，占地区生产总值比重从2000年的6.2％提高到9.7％。产业转型升级步伐加快，三次产业比重从2000年的8.7∶57∶34.3调整为5.2∶58.9∶35.9；石油化工、电子信息、旅游服务等产业有了新发展，汽车制造、修船造船、生物医药等产业实现新突破；规模以上工业产值占全市工业总产值的比重从2000年的32.2％上升到76.6％。金融业快速发展，2006年末人民币各项存款余额达1646.04亿元，各项贷款余额达1127.84亿元，分别比2000年末增加1033.97亿元和742.67亿元。县域经济快速发展，晋江、惠安、石狮、南安、安溪进入全国县域经济基本竞争力百强县（市），所有县（市）进入全省经济实力十强或经济发展十佳。

　　基础设施建设快步推进。累计完成固定资产投资2010亿元，超过前52年的投资总和。后渚大桥、大坪山隧道等重点项目建成投入使用，沿海大通道、金鸡拦河闸重建等项目基本建成，泉三高速公路（泉州段）、福厦铁路（泉州段）等项目动工建设；南埔电厂一期、晋江500千伏输变电等项目建成投入使用，全社会用电量从2000年的89.58亿千瓦时增至218.06亿千瓦时。固定电话、手机、互联网用户分别从2000年的133万户、104万户、12.5万户增至350万户、415万户、51万户。公路密度、高等级公路比率和公路硬化率走在全省前列，全市2057个、占99.85％的建制村实现通村公路硬化，率先在全省基本实现通建制村公路硬化目标。港口发展迈入新阶段，建成投产万吨级以上深水泊位12个，港口货物、集装箱吞吐量分别从2000年的1712.18万吨、15.89万标箱增加到2006年的5100万吨、83万标箱；泉州晋江机场客货吞吐量分别从2000年的36万人次、6547吨增加到2006年的115万人次、1.5万吨。

　　城市综合竞争力大幅提升。城市规划建设管理水平不断提高，规模不断扩大，中心城市建成区面积达80平方公里，比2000年增加40平方公里，进入全省三大中心城市行列。城镇发展步伐加快，全市城镇化水平比2000年提高12个百分点。城市公共服务设施大大改善，城市功能不断增强，中心市区建成区绿化覆盖率达39.76％，比2000年提高8.74个百分点。获国际花园城市、国家卫生城市、国家园林城市、最佳中国魅力城市、国家环保模范城市、水环境治理优秀范例城市和中国人居环境奖。

　　改革开放力度继续加大。先后出台实施进一步提升民营经济发展水平等一系列重大政策措施，民营经济进一步壮大，民营工业企业2006年总产值3165.6亿元，占全部工业总产值的90.8％；上市民营企业新增13家，全市百家重点企业中民营企业占98家。实施名牌发展战略，中国驰名商标、中国名牌产品分别增至72个和39个，居全国地级市前列，获中国品牌经济城市称号。深化行政审批制度改革，取消审批审核事项618项。169家国有工业企业实施改革，45家城镇集体企业完成改制。加快开放型经济发展，外经贸工作跃上新台阶，累计出口143亿美元，实际利用外资（报表口径）62亿美元，多家世界500强企业入驻泉州，泉州出口加工区通过海关总署等九部委验收并对外招商。

　　城乡统筹发展得到加强。贯彻落实中央三个“1号”文件，坚持工业反哺农业，城市支持农村，协调推进社会主义新农村建设，全市基本实现宽裕型小康目标。推进“4121”、“1168”等基地建设，建成特色农业产业基地122.2万亩，百家农业产业化龙头企业累计完成产值463.2亿元。取消农业税、农业特产税，累计减轻农民负担17.75亿元。加强农村公共服务，累计转移农村劳动力25万人；帮扶省、市级经济欠发达村140个，落实帮扶资金2.5亿元；完成“造福工程”搬迁4489户、19750人；新型农村合作医疗试点覆盖5个县（市、区）、77个乡镇、1266个建制村，参加人数达214.48万人，5.22万人获得住院补偿。有效防抗台风、洪涝、干旱等重大自然灾害。城乡人民生活水平有较大提高，农民人均纯收入从2000年的4440元增加到2006年的6606元，年均增长6.8％；城镇居民人均可支配收入从2000年的7817元增加到2006年的15972元，年均增长10.5％。

　　各项社会事业协调发展。大力推进科教兴市战略，组织实施五大国家级科技示范工程，获全国科技进步先进市“五连冠”。全面实施素质教育，“双高普九”取得明显成效，基本完成中小学危房改造3年计划，高中毛入学率达58.6％；教育现代化“八大工程”扎实推进，各类教育协调发展。华侨大学、泉州师院通过教育部本科教学评估，仰恩大学办学规模和质量进入全国民办本科院校前列，黎明大学获教育部高职高专院校人才培养工作水平评估优秀等级。泉州卫校等4所中专升格为大专。新增民办职业学院6所。人才强市战略深入实施，引进高级人才1358人，设立博士后科研工作站7个。文化事业加快发展，城乡文化设施和服务网络日趋完善，文艺精品创作取得丰硕成果，《董生与李氏》等27件作品获全国奖项。举办四届泉州旅游节。市县两级疾病控制、医疗救治和卫生监督体系建设基本完成。科学处置、有效防治“非典”、霍乱等重大疫情。探索建立人口计生工作新机制，继续稳定低生育水平，人口计生工作进入全省中上行列。群众性精神文明创建活动深入开展，获全国创建文明城市工作先进城市称号。

　　和谐社会建设成效凸显。社会保障体系逐步完善，城乡就业比较充分。城乡居民最低生活保障制度全面推行，纳入低保的人数和保障金发放从2000年的2.9万多人、1770万元增加到2006年的11万多人、7850万元。民主法制建设得到加强，“平安泉州”建设扎实推进，全面完成“四五”普法，启动“五五”普法，获全国法制宣传教育先进城市称号，实现全国社会治安综合治理优秀地市“二连冠”。加快少数民族地区发展，做好双拥、山海协作和援藏、援疆、援宁工作，获全国民族团结进步模范集体“三连冠”、全国双拥模范城“五连冠”。重视生态环境保护，狠抓晋江、洛阳江流域污染整治，人居环境明显改善。

　　与此同时，市十三届人大一次会议提出的1号、2号议案决议任务基本完成，市十三届人大四次、七次会议提出的两个议案决议正抓紧落实。六年来共办理市人大代表建议1762件、政协委员提案1986件，满意率分别达99.26％和99.28％。此外，67件130项为民办实事项目全部完成。

　　2006年是实施“十一五”规划的开局之年，全市按照市委的统一部署和“走前列、新突破”的要求，扎实做好经济社会各项工作，全市生产总值、财政总收入和一般预算收入分别比去年增长15.0％、20.6％和22.2％，迈出了“十一五”发展坚实的一步。

　　新农村建设稳步推进，“三农”工作取得新成效。出台推进社会主义新农村建设的实施意见，落实“五个一”措施，重点抓好省市县三级191个示范村和市级20个帮扶村工作。创新农村工作机制，11个县（市、区）完成“六大员”选聘配置工作。有效防抗“珍珠”、“碧利斯”、“格美”等台风和强暴雨，全力以赴抓好抗灾救灾和灾后重建工作。深入实施农业产业化“1333”工程，举办泉州龙眼（上海）展销会及第三届海峡两岸（泉州）农业合作交流洽谈会等展会。加强粮食储备和粮食安全体系建设。完善农村流通体系，建成农家店645家。抓好禽流感、牲畜口蹄疫及危险性林业有害生物等动植物疫病防控工作。完成“六千”水利、晋江下游防洪岸线整治等重点工程年度建设任务。推广农村沼气工程，开展“家园清洁行动”。完成“造福工程”搬迁5151人和150个50户以上自然村通广播电视任务。

　　项目带动战略持续深化，一批关系国计民生的重点项目投建。全社会完成固定资产投资490.75亿元，比增34.2％。泉州晋江大桥、泉州大桥南立交、外走马埭围垦等工程深入推进；福建炼化一体化、中化重油加工、石狮PTA项目进展顺利；泉州船厂修船项目万吨级造船区分段工程正式投产；一汽新华旭汽车项目、远东北方重工专业汽车项目、永春生物医药基地动工建设；8座220千伏变电站开工建设。项目对接成效明显，第四届“6·18”项目成果交易会实现对接项目491个，电子信息、石油化工、生物医药等新兴产业项目占30％。

　　企业自主创新力度加大，产业转型升级步伐加快。制定出台增强自主创新能力的一系列政策，组织实施科技项目200多项，市生产力促进中心先进制造技术服务基地建成投入使用，新增市级行业技术开发中心11家。加强知识产权保护，申请专利2000多件，登记版权制品5000多件。积极运用信息技术和先进适用技术改造传统产业，五大传统产业产值增长27.2％。大力培育发展“5＋1”新兴产业，五大新兴产业产值增长34.0％，旅游总收入增长20.8％。组织新兴产业重点企业赴成都、西安、哈尔滨等8个城市开展项目推介和高级急需人才招聘活动。推进节能降耗工作，重点抓好100家市级重点工业企业、66个省级工业经济新增长点项目和107个循环经济示范项目。

　　港城联动战略深入实施，城市发展空间进一步扩大。出台“十一五”期间加快港口发展意见，完成泉州港总体规划编制、泉州海洋功能区划修编。与中远集团建立全面合作关系，新开泉州港国际集装箱班轮航线。建成肖厝7万吨级散杂货码头，推进泉州湾中心港区深水航道疏浚拓宽等工程建设，完成港口建设投资8亿元。“一二三四”城建项目扎实推进。认真做好泉州市城市总体规划修编，完成泉州市域城镇体系规划、都市区协调发展规划、泉州市交通综合规划编制；东海、桥南、城东、江南等新区建设加快，朋山岭第二隧道全线贯通，城南片区（二期）道路、市区路面改造项目和钟楼等古城保护建设工作基本完成。房地产开发和监管得到加强。古城危房、城中村、城中厂改造有序推进。开通环泉州湾大公交线路。强化城市管理行政执法，制止“两违”取得阶段性成果。

　　外经贸支持服务体系不断完善，外向型经济进一步发展。建立健全出口预警和应对技术壁垒工作机制，抓好海关总署支持“海西”22条政策落实。全年外贸出口38亿美元（海关口径），比增21％。利用外资质量明显提高，新增投资超千万美元项目122个；外商实际到资（验资口径）9.2亿美元，比增29.8％。制定落实鼓励台商投资意见，组织企业赴澎湖商品展销，举办“鞋博会”、“海博会”、“海洽会”等经贸活动，实现泉澎货物直航，开通泉州港（石井）—金门航线，积极推进国家级台商投资区申报工作。与美国圣迭戈郡建立友好城市关系。做好重点侨情普查工作，进一步加强与海外侨胞的联络沟通。深化泉港合作八大平台建设，推进泉澳四项合作。组织企业参加“5·18”、“6·18”、“9·8”及广交会、中国—东盟博览会、深圳高交会等活动。

　　文化强市建设步伐加快，社会事业发展取得新成绩。中国闽台缘博物馆建成投入使用，11个项目入选首批国家级非物质文化遗产名录，梨园戏《节妇吟》和提线木偶《钦差大臣》入选国家舞台艺术精品工程提名剧目，《一轮明月》获中国电影华表奖。承办第六届福建省少数民族传统体育运动会，赴澎湖举办“泉州文化周”活动。加大教育支出，全年预算内投入教育经费达27.2亿元。认真贯彻新《义务教育法》，落实“两免一补”资金2.03亿元。积极发展中等职业教育，新增两所省级重点职校。落实高等教育发展规划，16所全日制普通高校在校生8.9万人。市第一医院新院动工建设，32家重点乡镇卫生院实现标准化目标，村级卫生所进一步完善。第六届全国农运会筹备工作进展顺利，“一场一馆”建设扎实推进，获第十三届省运会团体总分和金牌总数第三名。数字电视整体平移完成20万户。哲学社会科学、文学艺术、新闻出版、妇女儿童、老龄、未成年人、残疾人、档案、地方志、防震减灾、气象、民兵预备役、国家安全、海防、人防、口岸等工作取得新成绩。

　　和谐泉州建设持续加强，社会保持安定稳定。继续做好就业再就业工作。全市参加基本养老、失业、医疗、工伤和生育保险人数分别增至46万、41万、45万、35万和32万人。城乡低保人数覆盖面进一步扩大，享受低保人数新增5821人，发放保障金增加1250万元。深化企业养老保险制度改革，企业退休人员养老金人均月增加132元。出台贯彻《劳动法》意见，妥善处理劳动关系。认真贯彻党的民族政策和《宗教事务条例》，做好民族和宗教工作。重视信访工作，开展涉法涉诉接访活动，加强基层人民调解和法律服务。顺利完成村（社区）换届选举。深化平安创建活动，深入开展“打黑除恶”、“缉枪治爆收缴管制刀具”和打击“两抢一盗”等专项行动，继续实现“一升一降”目标。推进安全生产11个方面专项整治，事故起数、死亡人数、受伤人数继续下降。

　　近海水域等污染整治扎实开展，资源节约型和环境友好型社会建设取得成效。认真执行市十三届人大七次会议议案决议，制定实施《泉州湾南岸近海水域污染专项整治实施方案》、《泉州市近海水域环境污染综合治理方案》和晋江、洛阳江流域水环境保护与污染控制计划，落实“创模”持续改进措施，大力建设环保基础设施，全市投入近海水域污染整治资金12.62亿元，实施96个年度整治项目，已完成65个，晋江、南安、石狮、惠安污水处理厂建成或基本建成。加强山区生态林管护和自然保护区建设，新增造林面积18.3万亩。加强地质灾害治理和土地市场、矿业秩序整顿，实行最严格的耕地保护措施。推进土地集约利用，新建标准厂房685万平方米。完善人口计生工作责任体系，全年人口出生率10.19‰，政策符合率95.09％，完成省下达的人口计生控制目标。

　　政府自身建设得到加强，社会管理和公共服务水平进一步提高。以实施《公务员法》为契机，加强公务员队伍建设，推进机关效能和绩效评估工作。推行集中审批和网上审批，40个部门、481项审批服务事项入驻市行政服务中心运作。完善公共财政体制。加强国有资产监督管理。落实党风廉政建设责任制，加强审计监督和行政监察，开展商业贿赂专项治理。建立健全公共应急管理机制，出台应急预案57个，有效处置各类突发公共事件861起，政府行政能力和行政效能不断增强。

　　各位代表，过去六年，是我市经济实力持续增强、城乡面貌日新月异、人民群众生活水平明显提升的时期。成绩来之不易，这是省委、省政府和市委正确领导的结果；是全市干部群众团结一心、奋力拼搏的结果；是市人大、市政协大力支持和有效监督，各民主党派、工商联、无党派人士和离退休老同志关心支持，驻泉省部属单位、驻泉部队、武警官兵、公安干警，企业界人士、来泉务工人员和支持泉州现代化建设的港澳台同胞、海外侨胞、海内外朋友同心协力、共同奋斗的结果。在此，我代表泉州市人民政府向所有关心支持泉州发展的各界朋友和海内外人士致以崇高的敬意！对人大代表、政协委员和全市人民给予政府的高度信任和大力支持表示衷心的感谢！

　　在肯定成绩的同时，我们也清醒地看到，我市经济社会发展还存在一些困难和问题。主要是：人才、土地、资金、能源、原材料等生产要素制约日益凸显，发展压力进一步加大；受国际贸易摩擦、汇率变动及新一轮宏观调控影响，外贸出口面临新的挑战；产业升级步伐不快，传统产业自主创新能力不足；中心城市带动能力、辐射能力不强；第三产业发展不快，现代服务业总量偏小；城乡差距仍然较大，农村基础设施相对滞后，抵御自然灾害的能力还比较弱，农民增收长效机制还没有很好形成；城乡部分群众生活仍较困难；公共服务体系尚未健全，公共卫生、社会治安存在薄弱环节；安全生产形势不容乐观，不安全隐患还比较多；涉及群众切身利益的不少问题还没有得到很好解决，市区交通拥挤、环境污染、看病贵、特困群众住房难等问题反映仍较强烈；政府管理创新力度亟待加大，一些工作落实不够到位；部分公务人员法制意识、责任意识不强，行政不作为、乱作为现象仍有发生，依法行政水平有待提高；反腐败工作还需加强。此外，全市还有部分工作任务未达到预期目标，如2006年市《政府工作报告》确定的168项任务还有5项未完成。对此，我们要进一步采取措施，努力加以解决。

　　二、新一届政府工作目标和2007年政府工作

　　新一届政府的五年，是我市全面落实科学发展观、构建和谐社会、建设海峡西岸经济区现代化工贸港口城市的关键五年。我们要抢抓机遇，开拓创新，推动经济社会发展切实转入科学发展的轨道，努力实现速度、质量、效益相协调，消费、投资、出口相协调，人口、资源、环境相协调，真正做到又好又快发展。新一届政府工作的指导思想是：坚持以邓小平理论和“三个代表”重要思想为指导，以科学发展观统领经济社会发展全局，大力推进和谐社会建设，主动融入海峡西岸经济区建设大局，按照市第十次党代会总体部署，全面实施“十一五”规划，弘扬“三创”精神，落实“四个重在”要求，坚持科学、民主、依法行政，扎实推进社会主义新农村、经济强市、文化名城、和谐泉州四大建设，实现“五个基本”目标，发挥创业型城市经济快速发展的支撑和带动作用，持续走在全省发展前列。至2011年，预期全市生产总值年均增长11.5％以上，人均生产总值达到5000美元，一般预算收入年均增长16％以上，财政总收入突破350亿元，力争更好更快些。为此，新一届政府要认真贯彻中央和省委、省政府以及市委的决策部署，创造性地开展工作。

　　致力调整优化产业结构，转变经济增长方式。把整合提升传统产业和培育壮大新兴产业紧密结合起来，努力提高规模以上工业产值占工业总产值比重。加快五大传统产业集群化、信息化、品牌化进程，推动产业集聚、转型、升级，形成持续竞争优势。加快“5＋1”新兴产业的规划、培育、发展、壮大，延伸产业链，实现跨越式发展。抓住自主创新这个关键，把握集约发展这个根本，加大企业技术创新、成果应用和研发投入，依靠科技进步和提高劳动者素质，推动经济增长向集约型转变。

　　致力深化项目带动战略，增强经济社会持续发展后劲。坚持以项目促发展，围绕海峡西岸经济区现代化工贸港口城市建设和“十一五”规划，加强项目策划、储备、推介、论证，做细项目前期工作，生成和实施一批符合产业政策导向、带动力强、经济社会效益好的大项目。落实“五个一”工作机制，强化协调配合，加强管理服务，形成齐心协力推进项目建设的合力，用一批又一批关系长远、富有效益的好项目促进“十一五”规划的实施，增强经济社会发展的持续动力和后劲。

　　致力推进改革创新，激发发展活力。加快形成落实科学发展观的体制机制保障，稳步务实推进国有资产管理、行政管理、投资、财税及社会领域体制改革，力争在优化服务、提高效率、增强活力上取得新突破，努力消除影响科学发展、和谐进步的障碍。大力弘扬爱拼敢赢的泉州精神，营造鼓励人们锐意进取、大干事业的社会氛围，让一切劳动、知识、技术、管理和资本的活力竞相迸发，让一切创造社会财富的源泉充分涌流。

　　致力优化资源配置，保护生态环境。积极落实保护资源、保护环境的基本国策，贯彻节约发展、清洁发展、安全发展方针，大力发展“三高”、“三少”、“三节约”产业，坚决淘汰高消耗、高排放、低效益的落后生产能力，努力实现节能降耗和污染达标排放的约束性目标。加快发展循环经济，扎实做好节能、节水、节地、节材等工作，实现可持续发展，促进人与自然和谐相处，建设资源节约型、环境友好型社会。

　　致力促进社会和谐，保持社会安定有序。坚持以人为本，为民负责，利民惠民，着力解决好人民群众最关心、最直接、最现实的收入分配、就业、就医、住房、公共卫生、社会保障、社会治安、环境保护等问题，进一步关心和帮扶社会困难群体。积极培育和谐文化，大力推进精神文明和民主法制建设，不断增进干群关系和谐、劳动关系和谐、城乡关系和谐，建设一个公平公正、团结和谐的社会。

　　2007年是新一届政府的第一年，也是实施“十一五”规划的关键一年，我市经济社会发展主要预期目标为：地区生产总值增长12.5％以上；万元生产总值能耗下降3.5％；主要污染物二氧化硫和化学需氧量排放量分别减少4.2％和1.1％；财政总收入增长17％，其中一般预算收入增长17％以上；规模以上工业总产值增长22％；全社会固定资产投资增长20％以上；出口商品总值增长16％；实际利用外资（验资口径）增长10％；社会消费品零售额增长13％；居民消费价格总水平涨幅控制在3％以内；城镇居民人均可支配收入增长7.5％；农民人均纯收入增长7％；城镇登记失业率控制在1.5％以内，人口自然增长率控制在8.3‰以内。

　　（一）围绕新农村建设“走在前”，认真实施“五大工程”

　　一要实施规划工程。按照“生产发展、生活宽裕、乡风文明、村容整洁、管理民主”的总体要求，制定实施新农村建设五年规划纲要、泉州市村庄建设规划导则和乡村建设管理办法，完成10个乡镇总体规划修编调整。在巩固提升现有省市县三级示范村的基础上，确定培育一批试点示范村。认真做好第二次全国农业普查工作。二要实施发展工程。积极推进现代农业建设，提高农业水利化、机械化和信息化水平，提高农业素质、效益和竞争力。认真实施“1333”工程，加大“五新”推广力度，鼓励发展生态安全食品，组织开展茶叶生产许可认证，推进农产品品牌化和标准化。推广测土配方施肥。促进粮食稳定发展，提高粮食综合生产能力，完善粮食安全保障体系。提高泉州龙眼产业化、市场化水平，办好泉州龙眼（北京）推介会。抓好“万村千乡”市场工程，建立和改造农家店632家。发展海洋捕捞、海水养殖和水产品加工业，加快建设深沪、祥芝、崇武国家中心渔港。落实农村增收减负政策，转移就业6万人。加大山海协作和扶贫开发力度，实施“百村扶贫开发计划”，从项目、资金、政策上向安溪、永春、德化及“老、少、边、岛”地区倾斜，帮扶20个市级重点村。推进“造福工程”，搬迁安置5000人。三要实施基础工程。强化农村公共服务，加大农村公路维护提级改造、客运“村村通”和饮水安全工程的投建力度。推进农村信息化示范村和农村数字电视平移工作。推广农村沼气工程。完善农业防灾减灾体系，完成晋江下游防洪岸线整治及江滨路续建、山美灌区节水改造、“六千”水利工程和小山围塘除险加固年度建设任务，继续抓好碧坑水库项目前期工作。扎实推进非规划林地造林，强化森林防火工作。深入开展“家园清洁行动”和“小康环保行动”，重点解决26个镇（乡）、240个建制村的垃圾治理。四要实施素质工程。新增教育、卫生、文化等事业经费主要用于农村。深入开展“三下乡”活动，丰富农民文化生活。加快培育新型农民，不断提高农民群众适应工业化、城镇化和农业现代化的能力。组织实施劳动力创业、绿色证书等培训，完成培训5万人。做好省级乡镇“三农”服务中心试点，着力提高“六大员”素质和服务质量。深入开展文明村镇创建活动。五要实施保障工程。深化以税费改革为主要内容的农村综合配套改革，继续推进乡镇机构、农村义务教育和县乡财政体制改革，稳妥化解乡镇债务风险。推进林权配套改革。抓好3个市级土地承包权流转规范化试点，探索建立被征地农民社会保障制度。深化农村信用社改革，加强农村社会化服务组织建设。完成38个村级组织活动场所建设，确保全市所有村级组织“有址议事”。

　　（二）围绕打造先进制造业基地，加快产业转型升级

　　一要突出自主创新。认真实施增强自主创新能力的决定，以自主创新促进五大传统产业提升，市、县两级财政投入自主创新经费2.3亿元。鼓励行业协会和规模以上企业设立研发机构，新增省级以上工程技术研发中心、企业技术中心15家。充分利用省“6·18”平台，引导企业与科研院校建立长期科技协作关系。深入推进国家知识产权试点城市工作，设立知识产权发展专项资金，大力实施专利、标准和商标品牌战略，鼓励企业参与制定国家标准，新增国家级专利工作试点单位10家。推进科技资源整合共享，充分发挥高新技术企业孵化基地的作用，建成中小企业信息化、科技创业、知识产权3个公共服务平台。规划建设泉州软件园。大力推广新技术、新工艺、新材料应用，加快制造业信息化步伐。二要突出项目带动。认真实施126个在建重点项目，完成年度投资173亿元。坚持生产性项目先上，石油化工业重点推进福建炼化一体化、中化重油加工、石狮PTA项目；汽车制造业重点加快一汽新华旭、远东北方奔驰生产和锐驰汽车电子、深圳五洲龙项目开工；修船造船业重点加快泉州船厂修船项目建设，争取列入国家规划的大型造船项目启动；电子信息业重点建设微波射频、光伏电子、手机通讯制造项目；生物医药业重点抓好永春生物产业基地和华宝、海裕、海汇、海鑫等生物科技项目；同时认真抓好100家市级重点工业企业、57个省级工业经济新增长点项目。坚持商贸性项目先上，重点建设石狮服装城二期、泉州晋江铁路综合货场、石湖港石材物流交易市场、南安官桥粮食城、泉州电子汽车汽配城等项目。坚持与国计民生密切相关的重大基础设施先上，抓好泉三高速公路（泉州段）、福厦铁路（泉州段）、外走马埭围垦等项目续建，争取泉三高速南安至惠安支线、沈海高速复线南安金淘到厦门高速公路开工，做好泉州湾跨海大桥等项目前期工作。三要突出提升服务业。围绕建设旅游强市，整合旅游资源，完善旅游设施，建设南少林寺旅游区等项目，发展文化旅游、滨海旅游、生态旅游、红色旅游。加快发展现代服务业，着手规划城市商务集中区，抓紧江南新区商贸物流中心、城东片区购物中心及东海片区现代商贸区的规划建设，培育壮大金融保险、电子商务、法律服务、信息咨询业，鼓励发展工业设计、市场推广、产品检测、售后服务等生产性服务业。四要突出要素保障。深化人才强市战略，继续实施“112专家工程”、“222高技能人才工程”，精心组织2~3场赴省外招聘中高级急需人才活动，培育自主创新人才队伍。加强用工、资金、用地保障，办好第三届企业劳务洽谈会、第五届银企资金供需洽谈会，盘活闲置土地，推进标准厂房建设。加强电力保障，推进2个500千伏、12个220千伏、54个110千伏变电站建设，抓好南埔电厂二期、晋江燃气电厂和石狮鸿山热电厂等电源项目建设。

　　（三）围绕建设现代化亿吨大港，增强港口综合竞争力

　　一要统筹港口发展。认真实施“以港兴市、港城联动”战略，大力推进泉州新港和湄洲湾南岸主枢纽港建设，确保2007年港口货物吞吐量6200万吨、集装箱突破100万标箱。积极争取《泉州港总体规划》获批，编制完成重点作业区控制性详规。大力推进秀涂码头、石湖10万吨级码头、围头防波堤技改工程、斗尾30万吨级原油码头、肖厝10万吨级码头等项目建设，加快疏浚拓宽泉州湾、围头湾、湄洲湾三条通海航道，争取年度投资10亿元。抓好湄洲湾交管系统、泉州海巡基地、溢油应急反应中心建设，确保航运安全。完善集疏运体系，推进漳泉肖铁路肖厝支线建设，拓展港口腹地。二要壮大港口经济。依托港口优势，大力发展石化、汽车、修造船、机械等临港工业。抓紧编制港口物流发展规划，积极吸引船公司和大型船货代公司来泉设立物流公司，启动中远石湖物流园区建设，壮大港口物流业。争取石湖作业区设立出口监管仓库，开辟近远洋支线，增加外贸集装箱量。引导航运公司整合重组，积极引进外代公司，加快航运业规模发展。三要营造良好环境。设立港口发展专项资金，认真落实港口经营、建设、外贸运输和货物出口等优惠政策，大力培养和引进港口物流、航运等专业人才。创新港口投入机制，研究实施“地主港”经营模式。深化港口管理体制改革，集中港口行政事权，提高管理效率。加强“大通关”建设，努力实现“一站式”、“全天候”、“一条龙”通关服务。

　　（四）围绕办好全国农运会，提升城市规划建设管理水平

　　一要拓展城市空间。以举办2008年全国农运会为契机，全力推进“一二三四”城建项目建设。城东片区要加快形成以中心大道、江滨路为纽带的主次干道网络，确保“一场一馆”竣工、滞洪排涝工程基本建成和安置房一、二期主体封顶，初步形成片区现代城市景观。东海片区要加快推进迎宾馆、综合大道、东西景观路、滨海大道、东海围垦等项目。桥南片区要重点抓好池峰路、兴贤路等4条主干道施工，完成80％安置房建设任务，开工建设市民广场及周边商住楼群。建立都市区协调发展机制，实施晋江、洛阳江两岸和沿海大通道两侧城市景观带规划，统筹都市区基础设施、功能定位、产业布局，加快发展泉州湾沿岸的城市核心地带。二要完善城市功能。优先发展公交，新建江南、丰泽公交车站。优化城市路网，确保泉州大桥南立交、旧泉永路竣工通车，加快泉州晋江大桥、市区内环路建设步伐，深入推进一、二环路前期工作，做好泉州铁路综合枢纽站规划，增强中心市区交通枢纽功能，有效缓解城市交通压力。加快实施燃气工程项目，推进泉州金浦水厂建设，进一步提高市政设施的承载力。推进古城区危房整治、学府路、南少林片区、田安路南拓和西街保护等项目，完成温陵路、九一街与打锡街、国道324线城东段等街道立面改造。三要强化城市管理。深化城市管理行政执法体制改革，坚决制止“两违”，深入开展中心市区交通整治，强化城市卫生管理，抓好市容环境综合治理，提高市民文明素质。

　　（五）围绕深化改革开放，增强全球化参与能力

　　一要深化经济体制改革。完善国有资产监督管理体制，加快推进国有和城镇集体企业改革，加快国有资产战略性调整，组建运输集团和城建集团，壮大港务集团，深化中侨集团改革。创新投融资体制，探索项目代建制、业主招标等模式，鼓励社会资本、境内外战略投资参与城市基础设施、污水垃圾处理、公交等领域建设经营。鼓励企业改制上市。规范发展中介组织，促进部分政府职能依法授权或委托具备条件的行业协会承担。二要提高经济国际化水平。突出招商选资，促进在谈项目落地投产，力争在引进世界500强企业、跨国公司投资高新技术产业、先进制造业、现代服务业和建立研发中心、采购中心等方面取得新突破。高标准做好泉州出口加工区、洛秀组团项目招商。提升“海博会”、“鞋博会”、“网洽会”、“石博会”、“海洽会”等重大会展的影响力。健全出口风险防范应对机制，优化出口产品结构、市场结构和外贸主体结构，引导企业积极应对国际贸易摩擦和鞋、服装、家具等反倾销。支持优势企业“走出去”，到境外建立营销机构、贸易网点和投资设厂，推动企业参与境外经济贸易合作区建设。三要密切台港澳侨合作。落实鼓励台商投资政策，积极跟踪台商投资区申报，完成海峡两岸（泉州）农业合作试验区规划，办好惠安台商创业基地、海峡两岸农产品物流中心，主动承接台湾石化、机械、电子、现代农业等产业转移。继续深化泉港八大合作平台建设和泉澳四项合作，争取在企业合作、人才交流、贸易往来、联合招商等方面取得新进展。做好侨务工作，努力解决华侨农场改革后存在的困难和问题，维护归侨侨眷合法权益，扩大与海外侨亲和华人社团的联谊。实施“回归工程”，鼓励和吸引海内外泉籍企业家参与家乡建设。

　　（六）围绕建设文化强市，大力发展各项社会事业

　　一要坚持教育优先发展。进一步加强素质教育，提高各级各类学校的教育质量。全面实行对县（市、区）政府教育工作的督导评估制度，新增惠安、德化两县实现“双高普九”。加快教育资源整合，稳妥做好中小学布局调整，办好市区公办初中学校；推进优质教育资源与农村薄弱学校对口帮扶，扶持改造20所农村薄弱学校，扶持15所农村寄宿制小学配套建设，扶持5所公办中等职业学校实训基地配套建设。加快普及高中阶段教育，高中毛入学率达62％。认真执行农村义务教育阶段“两免一补”政策，依法保障城镇贫困家庭和进城务工人员子女平等接受义务教育权利，帮扶城乡贫困学生就学。发展中高等职业教育，继续支持办好辖区内高等院校。依法规范民办教育。重视学前教育、成人教育、老年教育和特殊教育，建设学习型社会。二要加强医疗卫生服务。进一步加强疾病预防控制和公共卫生监督体系建设，提高卫生应急处置能力。大力发展城市社区卫生服务，中心市区基本建立社区卫生服务体系。实施农村卫生服务体系建设与发展规划，新增洛江、南安、惠安、永春为新型农村合作医疗试点，新增受益人口160万人。加快推进市第一医院新院、泉州医疗中心（一期）及福建医大附属二院新院建设，推进22所重点乡镇卫生院标准化建设。整顿医疗服务市场和药品流通市场，规范医疗执业行为，努力降低医疗费用。三要进一步繁荣文化事业。积极稳妥推进文化体制改革，加快发展文化事业和文化产业。推进“海丝”申报“世遗”和“泉州南音”申报“人类口头及非物质遗产代表作”，抓好民族民间文化保护工程综合性试点。大力弘扬闽南文化，发挥中国闽台缘博物馆平台作用，加强泉台文化交流。完成梨园古典剧院、南音艺苑等项目建设，加快泉州木偶剧场动工，推进东西塔保护和承天寺景观修复工程。加强文化先进县创建工作，建立和完善乡镇、村（社区）文化网络，丰富城乡基层群众和企业文化生活。加强文化人才队伍建设，培育一批优秀文艺人才。深入贯彻妇女儿童发展纲要，依法保障妇女儿童合法权益，促进妇女儿童全面发展。四要加快发展体育事业。坚持“节俭办农运”，倒计时做好2008年全国农运会各项筹备工作。深入开展群众性体育活动，办好第八届市运会，加强老年健身工作，提高群众健康水平。五要提高人口计生水平。健全人口计生工作新机制，稳定低生育水平，提高人口素质，推行优生优育，综合治理出生人口性别比偏高问题，加强流动人口计生管理和服务，不断提高人口和计划生育依法行政、社会管理、公共服务水平。六要促进双拥工作再上新台阶。做好国防动员、民兵、预备役工作，落实各项优抚政策，妥善安置退伍、转业军人和随军家属，积极支持部队建设，争创全国双拥模范城“六连冠”。

　　（七）围绕构建和谐泉州，增进社会团结和睦

　　一要培育和谐文化。推进社会主义核心价值体系建设，大力弘扬社会主义荣辱观，扎实开展群众性精神文明创建活动。倡导见义勇为，弘扬社会正气。开展社会公德、职业道德、家庭美德教育，加强政务诚信、商务诚信、社会诚信建设，促进人际和谐。二要发展和谐劳动关系。全面落实各项促进就业政策，抓好统筹城乡就业试点，健全再就业援助制度，进一步解决我市用工结构性矛盾，全年培训各类劳动力25万人，新增就业15万人。健全劳动关系三方协调机制，推进“企业劳动合同三年行动”，确保工资按时足额发放。认真做好辖区内大中专毕业生就业指导和服务工作。三要完善社会保障制度。有效增加中低收入家庭的住房供应，加快江南、见龙亭及各县（市、区）经济适用房和廉租房建设。健全“五险合一，地税征收”机制，继续提高社保参保率。深化养老保险制度改革，完善机关事业单位养老保险制度。提高城乡低保标准，扩大农村低保覆盖面。全市参加基本养老、失业、医疗、工伤、生育保险分别达49.7万、45万、50万、40万和35万人以上。加快发展福利、慈善事业，构建覆盖城乡的新型社会救助体系，提高重点优抚对象、“五老”人员、“五保户”和残疾人、孤残儿童等特殊困难群体的福利水平。四要促进人与自然相和谐。认真执行市十三届人大七次会议议案决议，深入抓好泉州湾近海水域污染治理和全市近海水域污染综合治理方案落实，继续推进晋江、洛阳江流域环境综合整治，建立“两江”上游生态公益林建设补助机制，抓紧制定泉州市水资源配置调度管理规定。建成安溪、德化污水处理厂，基本建成北峰污水处理厂，加快城市污水管网建设。完善县、乡（镇）、村垃圾收集转运处理三级管理体系，集中式饮用水水源水质达标率96％以上，近海水域水质功能区达标率50％。合理开发利用矿产资源，加大对耕地的保护和管理，加强对国道、省道及“两江”沿岸“青山挂白”的治理。严格实施海洋功能区划，科学开发海洋资源。加强对重点耗能行业和企业的节能监督管理，抓好对100家国家、省、市跟踪的重点耗能企业的节能督导。落实55个循环经济示范项目，推行清洁生产和ISO14000环境管理体系认证，大力发展循环经济。五要深化“平安泉州”建设。深入开展社会治安综合治理，完善基层服务和管理网络，提高排解社会矛盾纠纷能力，减少社会群体性事件发生。健全信访工作责任制，提高信访工作水平。严厉打击涉黑涉恶和暴力犯罪、盗窃、抢夺等多发性侵财犯罪及“六合彩”赌博、信息诈骗等违法犯罪活动，深入开展禁毒人民战争。进一步落实安全生产责任制，继续抓好11个重点行业和重要领域安全专项整治，建立市级重大危险源监控系统，严格事故责任追究，坚决遏制重特大安全事故发生。

　　三、建设一个服务发展、促进和谐的创新型政府

　　今后五年，新一届政府要强化以人为本、执政为民的理念，加强自身建设，推进政府管理创新，提高行政能力和管理水平，努力建设法治政府、服务政府、责任政府和效能政府。

　　创新管理体制，进一步增强政府的保障力。深化行政管理体制改革，优化机构设置，全面履行经济调节、市场监管、社会管理和公共服务职能。创新经济管理方式，运用经济和法律手段，深入整顿规范市场秩序，加强食品安全监管，严厉打击侵犯知识产权、制假售假等违法行为。创新社会管理，加强基层自治组织建设，健全党委领导、政府负责、社会协同、公众参与的社会管理格局。创新公共服务体制，加强公共设施建设，促进城乡基本公共服务均等化。完善突发公共事件应急管理机制，提高快速反应和综合控制能力，保障公共安全。

　　创新运作机制，进一步增强政府的执行力。健全科学民主依法决策机制，认真落实市政府工作规则，坚持重大事项集体研究决定。加强决策督查反馈，定期对决策执行情况进行评估。贯彻《公务员法》，深化机关效能建设和绩效评估工作，形成“想干事、能干事、干成事”的激励机制。建立行政问责制，有效解决不作为、乱作为和有令不行、有禁不止问题，确保政令畅通。认真落实全面推进依法行政实施纲要，深入开展“五五”普法，完善行政执法责任制和行政复议、行政赔偿制度，严格依法行政。

　　创新服务方式，进一步增强政府的公信力。深化行政审批制度改革，完善集中审批和网上审批，推行并联审批，开通“绿色通道”，推进市、县、乡三级审批联动，进一步优化投资软环境。深化政务公开，在与群众利益密切相关的部门全面推行办事公开制度，扩大公开范围和层次，提高政府的透明度和诚信度。拓宽社情民意表达渠道，健全领导干部接待群众制度，完善“政风行风直通车”、“一线通”、“行风热线”等沟通平台，努力解决群众反映的热点难点问题。认真办好市委、市政府为民办实事项目，取信于民。

　　创新监管制度，进一步增强政府的约束力。认真贯彻《监督法》，自觉接受监督。全面落实党风廉政建设责任制，健全教育、制度、监督并重的惩治和预防腐败体系。强化审计监督和行政监察，加大对行政自由裁量权、建设工程项目招投标、经营性土地使用权出让、产权交易、政府采购等领域的监督力度。惩防并举，加大商业贿赂治理力度，大力纠正行业不正之风。牢记“两个务必”，谦虚谨慎，艰苦奋斗，勤俭办一切事业。

　　新一届政府肩负着泉州发展的重任，肩负着全市762万人民的重托。我们一定要在市委领导下，自觉接受市人大及其常委会的法律监督、工作监督，完善向市人大及其常委会报告工作的制度，执行市人大及其常委会的决议、决定，支持市人大开展执法检查；自觉接受市政协和各民主党派的民主监督，积极支持政协履行政治协商、民主监督和参政议政职能，认真听取各民主党派、工商联、无党派人士以及专家学者的意见，及时办理人大代表议案、建议和政协委员提案；自觉接受新闻媒体和人民群众的监督，发挥工会、共青团、妇联等群团组织作用，共同推进泉州改革开放和现代化建设的伟大事业。

　　各位代表！泉州的发展站在一个新的历史起点上。让我们紧密团结在以胡锦涛同志为总书记的党中央周围，高举邓小平理论和“三个代表”重要思想伟大旗帜，全面落实科学发展观，构建和谐社会，在市委领导下，充分发挥全市人民的积极性、主动性和创造性，同心同德，开拓创新，为实现“十一五”规划的宏伟目标，为建设海峡西岸经济区现代化工贸港口城市而努力奋斗！
